# Juxtacaval Liver Resections With the Use of an Internal Ivc Shunt Tube

**DOI:** 10.1155/1990/97192

**Published:** 1990

**Authors:** Yoshiyuki Shimamura, Peter Gunvén, Masanori Ishii, Hiroshi Hasegawa

**Affiliations:** 1 National Matsudo Hospital Matsudo Chiba Japan; 2 National Cancer Centre Hospital Tokyo Japan; 3 Department of Surgery National Matsudo Hospital 123-1 Takatsukashinden Matsudo Chiba 271 Japan

## Abstract

Almost one tenth of more than 370 hepatectomies, mostly for tumors, involved resection of major parts of the caudate lobe, subsegment 1. Five of them were for tumors or hemangiomas here, compressing or invading the vena cava; two were for metastases of colorectal cancer located very close to the junctions of the right and middle hepatic veins with the vena cava. We would previously have deemed these tumors unresectable. In these patients the vein was banded above and below the liver, an internal shunt tube placed in preparation for shunting of blood, and the afferent liver blood flow controlled. Control of the vena cava by tightening of the bands was needed in two cases. Tumor-invaded parts of the vein wall were resected in two other cases, in whom the presence of the tube facilitated the resection but the bands did not have to be tightened. The procedure did not cause morbidity and we conclude that tumors close to the vena cava can often be resected without complex vascular exclusion techniques, even when they invade the vein.


*HPB Surgery* 1990, Vol. 2 pp. 121–127
Reprints available directly from the publisher
Photocopying permitted by license only

© 1990 Harwood Academic Publishers GmbH
Printed in the United Kingdom

### JUXTACAVAL LIVER RESECTIONS WITH THE USE OF AN INTERNAL IVC SHUNT TUBE

YOSHIYUKI SHIMAMURA, PETER GUNVÉN, MASANORI ISHII, and HIROSHI HASEGAWA

*National Matsudo Hospital, Matsudo, Chiba, Japan and the National Cancer Centre Hospital, Tokyo, Japan*

*(Received 17 January 1989)*

Almost one tenth of more than 370 hepatectomies, mostly for tumors, involved resection of major parts of the caudate lobe, subsegment 1. Five of them were for tumors or hemangiomas here, compressing or invading the vena cava; two were for metastases of colorectal cancer located very close to the junctions of the right and middle hepatic veins with the vena cava. We would previously have deemed these tumors unresectable. In these patients the vein was banded above and below the liver, an internal shunt tube placed in preparation for shunting of blood, and the afferent liver blood flow controlled. Control of the vena cava by tightening of the bands was needed in two cases. Tumor-invaded parts of the vein wall were resected in two other cases, in whom the presence of the tube facilitated the resection but the bands did not have to be tightened. The procedure did not cause morbidity and we conclude that tumors close to the vena cava can often be resected without complex vascular exclusion techniques, even when they invade the vein.

KEY WORDS: Hepatectomy, vena cava, caudate lobe

### INTRODUCTION

The caudate lobe of the liver lies to the left of the inferior vena cava (IVC) with a caudate process extending to the right, ventral to the vein. This conventional description disregards the surgically important portal anatomy, that defines what has been called segment or subsegment 1 (S1)[1] or segment dorsale[2]. The study of liver casts[3] and intraoperative ultrasonography (US) with intraportal dye injection for regional staining of the liver [4] have improved the knowledge of the anatomy of S1. The segment usually receives several branches from both lobar portal triads so that we can speak of a right and left S1.

Resections of the right lobe often include large parts of the right S1 without technical problems. However, removal of major parts of S1 close to the IVC, particularly the left S1 in the presence of pathological changes, is treated with respect in texts on liver surgery[2,5,6]. Multiple short vessels between S1 and the IVC or portal vein, abnormal anatomy and inflammation may contribute to the difficulties. Tearing of the IVC has led to operative deaths[6–9], even during resection for benign disease[7]. Vascular isolation of the liver[5,8,10] to avoid the problems is complicated, possibly gives excess mortality, and has been abandoned by some of its users[11]. Recomended preparatory resection of

Correspondence to: Dr. Yoshiyuki Shimamura, Department of Surgery, National Matsudo Hospital, 123–1 Takatsukashinden, Matsudo, Chiba 271, Japan.

the left lateral segment[2] or of the entire left lobe[6] to improve access to S1 may be impracticable due to liver dysfunction.

We have used a relatively simple method for control of the afferent liver blood flow and of the intrahepatic IVC or supra- and infrahepatic banding around an internal shunt tube within this vein to improve resectability of lesions in subsegment 1.

### PATIENTS AND METHODS

*Patients.* Thirtythree of more than 370 hepatectomies between 1980 and 1988 involved resection of major parts of S1. The shunt tube was devised in 1985 and used in five of them, and in two patients with tumor close to the junctions of the right and middle hepatic veins with the IVC. Clinical features of the patients are given in Table 1.

*Preoperative Imaging.* Routine diagnostic imaging consisted of selective or superselective liver arteriograms, arterial portograms, enhanced CT scans, and US[12]. Magnetic resonance imaging (MRI) was informative in three recent cases (Figure 1). Cavograms were obtained if any imaging modality demonstrated compression of the IVC; abrupt or irregular narrowing of the lumen suggested neoplastic invasion of the wall.

*Operative Technique.* The incision in the form of an inverted asymmetric T or a J started in the mamillary plane, included xiphoidectomy, and was extended as needed. The liver was extensively mobilized before intraoperative US. The infrahepatic IVC was banded above the renal veins and the suprahepatic vein below the diaphragm, or in the pericardial space after sternotomy. A purse string suture was placed in the ventral wall of the infrahepatic IVC, if possible above the renal veins, a curved vascular clamp applied, and the shunt tube inserted via a phlebotomy (Figure 2).

The shunt tube (Figure 3; not commercially available) of heparin-coated PVC had inner/outer diameters of 6/8 mm. A side hole on its caudal convexity inside the IVC admitted blood to its lumen. The suprahepatic band encircled the IVC around the cranial end of the tube, and the infrahepatic band similarly around the tube cranial to the side hole, where two ridges on the outer surface fixed the tube and identified the location of the side hole. The protruding caudal end of the tube was clamped and regularly flushed with heparin-saline. A stylet with two balls occluded the tube lumen on both sides of the side hole during insertion and withdrawal of the tube to decrease blood loss.

**Table 1** Clinical features of patients in whom an IVC shunt tube was placed*

| *Case No.* | *Age, sex* | *Diagnosis* | *Size of lesion (cm)* | *Subsegmental location* |
|---|---|---|---|---|
| 171 | 37, M | HCC | 10 × 15 | S1 |
| 202 | 52, F | hemangioma | multiple | rt lobe-S1 |
| 264 | 61, M | met. of colon cancer | 8 × 12, 5 × 6 | rt. lobe-S1 |
| 281 | 59, M | met. of rectal cancer | 4 × 7, 2 × 3 1 × 1, 3 × 3 | S3 + 7 + 8 + 8 |
| 282 | 53, F | HCC | 7 × 8 | S1 + in hepatic v. |
| 304 | 72, M | met. of colon cancer | 7 × 9, 7 × 8 | S8-4 near hepatic vv. |
| 316 | 53, F | hemangioma | 9 × 12 | S1 |

*subsegments numbered according to ref. 1: S1 rt + lt caudate lobe, 3 lt ventral lateral, 4 lt medial, 7/8 rt postero-/anterosuperior. HCC hepatocellular carcinoma.

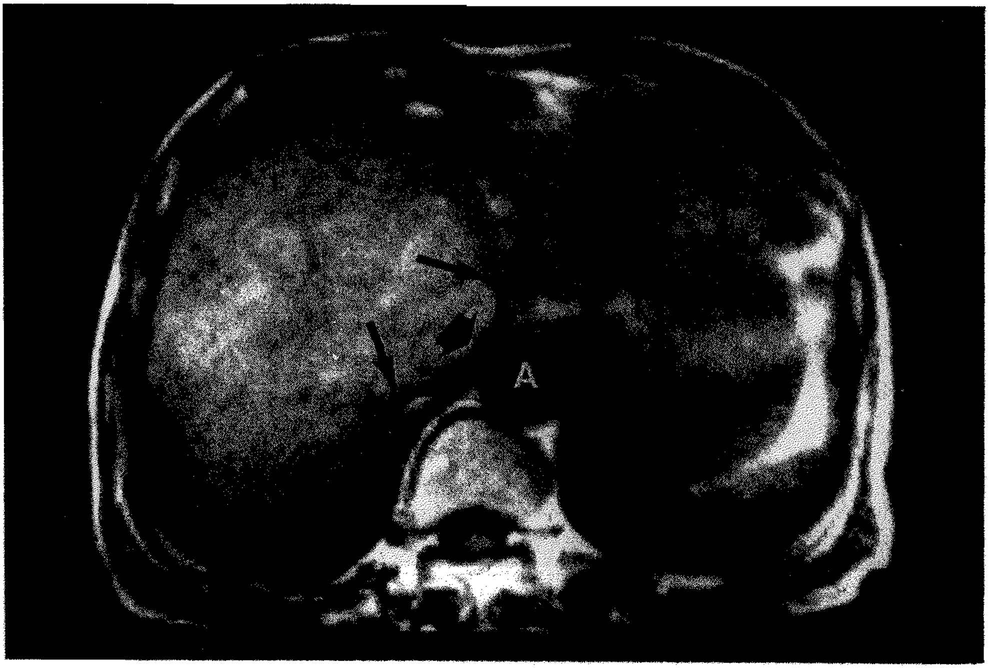

**Figure 1** MRI of the liver of patient no. 304. a large metastasis (high signal area) between the right and middle hepatic veins, indicated by slender arrows, compresses the IVC (broad arrow). A = aorta.

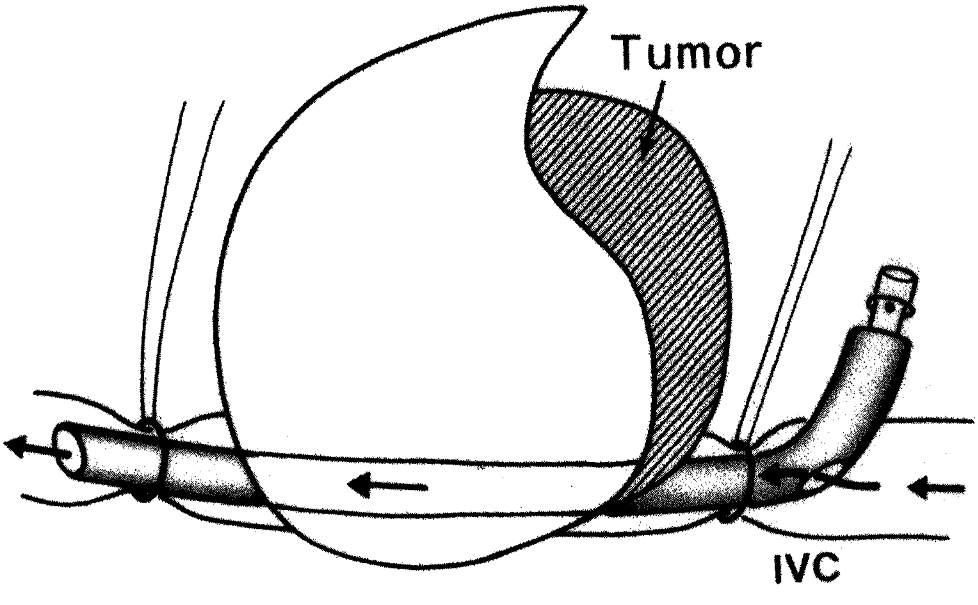

**Figure 2** Schematic drawing of the shunt tube in place. Constriction by the bands excludes the intrahepatic IVC from the blood flow from the caudal IVC, that enters the shunt tube via the caudal side hole.

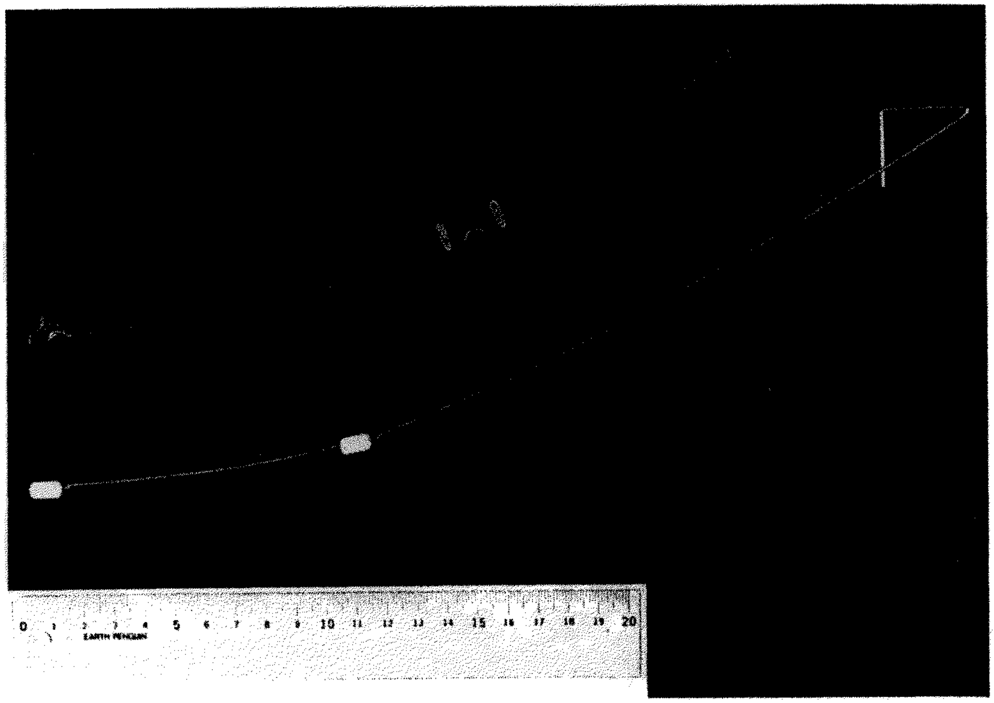

**Figure 3** Present version of the shunt tube with its caudal end to the right and the occluding stylet used during insertion and removal of the tube below.

The inferior phrenic vessels were occasionally divided but the right adrenal vein was ligated in only one instance, during resection of a tumor-invaded adrenal gland. To control the intrahepatic IVC, the bands were tightened around the shunt tube and the afferent liver vessels clamped. The liver was dissected from the IVC with ligation of the short hepatic veins and an ultrasonic aspirator was used to divide the parenchyma. Individual details are given in Table 2.

### RESULTS

*Operations Using The Shunt Tube.* Two benign and three malignant tumors in S1 close to the IVC, and two cases of metastases in the right anterosuperior subsegment ($S8^1$) close to the IVC and the middle and right hepatic veins were removed after placement of the shunt tube (Tables 1–2). The intrahepatic IVC was kept patent by the tube in all cases and the bands were tightened in two of them. Part of the intrahepatic IVC wall could be resected with the use of longitudinally placed vascular clamps without exclusion of the vein in two other cases. The clamps then constricted the vein around the tube that maintained an open lumen, shunting blood in a non-excluded IVC.

All resections were macroscopically radical except one with exclusion of the IVC, case no. 171. Here, the margins were questionable at the wall of the vein,

**Table 2** Operative details when an IVC shunt tube was placed

| *Case No.* | *Extent of resection* | *Technical details* |
|---|---|---|
| *171* | *extended lt lobectomy + S1* | *sternotomy, rt adrenalectomy, IVC freed from dorsal abdominal wall. 16 hrs operating time, 20 min. shunting, 5.3 l blood loss* |
| *202* | *extended rt lobectomy* | *resection of IVC wall 0.7 × 5 cm using clamps. 8 hrs operating time, 2.5 l blood loss* |
| *264* | *rt trisegmentectomy* | *sternotomy, rt adrenalectomy, resection of diaphragm. 9 hrs operating time, 4.2 l blood loss* |
| *281* | *subsegment 3 + 3 enucleations* | *resection of invaded stomach. 6 hrs operating time, 25 min. shunting, 1.4 l blood loss* |
| *282* | *extended lt lobectomy + S1* | *resection of IVC wall 1 × 2 cm. 9 hrs operating time, 1.8 l blood loss* |
| *304* | *lt medial + rt ant. segments* | *resection of diaphragm. 7 hrs operating time, 0.9 l blood loss* |
| *316* | *lt lobectomy + entire S1* | *IVC freed from dorsal abdominal wall, completely surrounded by hemangioma. 7 hrs operating time, 2.0 l blood loss* |

histopathology was inconclusive but at autopsy four months later this area was free from tumor.

*Bleeding And Hemodynamic Consequences.* The range of operative blood loss was 860 – 5,300 ml with a mean of 2,600 ml. The largest loss occurred in a patient with adhesions after previous exploration.

The shunting had mild hemodynamic consequences. In two patients in whom the bands were not tightened but vascular clamps occluded the IVC lumen around the shunt tube, blood pressure and pulse rate did not change. Shunting induced by constriction of the bands in patient no. 171 led to a decrease of blood pressure from 100 – 130 mm Hg to about 100 after ten minutes, after which a slight head-down position quickly restored it to 120. In the other patient with IVC exclusion and shunting, no. 281, the systolic blood pressure fell from 135 to 90 during the first ten minutes with a simultaneous increase of pulse rate from 90 to 105 per minute. This condition was stable during 15 more minutes of exclusion. It was retrospectively found that the caudal side hole of the shunt tube had been far advanced so that it was occluded by the IVC wall, thereby preventing shunting of blood.

*Morbidity And Mortality.* No postoperative complications attributable to the shunt tube occurred. One hospital death 1 1/2 months postoperatively was due to liver failure following right trisegmentectomy after transarterial liver embolization and chemotherapy. The other hospital death, at four months, was also due to liver failure after an extended left lobectomy followed by arterial chemoembolizations with Lipiodol—doxorubicin—gelatin sponge cubes[13]. Autopsy of the patient revealed tumor growth in the liver remnant but not at the IVC, as mentioned above. The other patient did not have macrosocopical tumor growth at autopsy.

*Long Term Results.* The two patients with hemangiomas were symptom-free 14 and 36 months after operation. One of the patients with metastases of colorectal cancers is alive with liver recurrence at 22 months, the other died free from tumor due to gastrointestinal bleeding after more than 6 months. One patient with HCC died from bone metastases more than 10 months postoperatively.

### DISCUSSION

Many liver resections include parts of S1 without problems, but juxtacaval tumors may be difficult or impossible to resect. Even thorough preoperative imaging by US, CT, MRI and cavography may not give reliable information about invasion of the IVC wall, that in itself does not preclude resection, according to our experience. We therefore explore potentially resectable tumors but have felt that a simple method for control of the intrahepatic IVC was desirable if the vein was operatively damaged or had to be resected. The control should be easy and rapid to apply, and shunting of blood should maintain circulatory stability.

Several methods for such exclusion of the IVC with shunting have been devised[8,10, reviews: 1,5,14,15]. Our technique combined features of several others but seemed to be one of the simplest. No complications attributable to the placement and use of the shunt tube were observed and the hemodynamic consequences were mild or absent.

Theoretically, the control was incomplete because phrenic vessels, the right adrenal vein, and hepatic veins receiving blood via aberrant uncontrolled hepatic arteries may empty into the excluded segment of the IVC. This, and the time limit set by the permissible duration of warm liver ischemia during control of the afferent liver blood flow, did not pose practical problems. In patient no. 171 the dissection gave small defects in the IVC wall but these gave negligible bleeding. In this patient, however, the right adrenal vein was ligated.

Even when the IVC is not excluded, the presence of the tube may be of value. Tearings of the IVC occurred in all cases and were easy to control by pressure against the tube before tightening of the bands and suture. In two cases in whom part of the IVC wall was resected with the use of vascular clamps, the tube maintained an open lumen for the passage of blood.

We placed the shunt tube in seven of about 200 hepatectomies after it was devised in 1985. It can therefore be estimated to be desirable in about five percent of our resections. This figure may not be generally valid because our unit is a secondary referral center; thus two of the patients in this series came from the U.S. and Australia.

### *Acknowledgments*

Supported by grants from the Japanese Foundation for Promotion of Cancer Research, the Japanese Government's Ten-Year Strategy Against Cancer, the Swedish Cancer Society, and the Sasakawa Foundation.

### *References*

1. Couinaud, C. (1981) Controlled hepatectomies and exposure of the intrahepatic bile ducts. Anatomical and technical study. Paris: C Couinaud.
2. Tung, T.T, (1979) Les résections majeures et mineures du foie. Paris: Masson & Cie, Editeurs.

3. Kumon, M. (1985) Anatomy of the caudate lobe with special reference to portal vein and bile duct. *Acta Hepatologica Japonica*, **26**, 1193–1199.
4. Makuuchi, M., Hasegawa, H., & Yamazaki, S. (1985) Ultrasonically guided subsegmentectomy. *Surgery Gynecology and Obstetrics*, **161**, 346–350.
5. Bourgeon, R. & Guntz, M. (1975) Foie and voies biliaires intrahépatiques. In: Patel, J., Leger, L., eds. Noveau traité de technique chirurgicale. Tome XII, Fascicule premier. Paris: Masson & Cie, Editeurs, pp 95–108, 195–196.
6. Foster, J.H. & Berman, M.M. (1977) Solid liver tumors. In: Ebert, P.A., ed. Major problems in clinical surgery, vol. **22**. Philadelphia: WB Saunders Co., pp 257, 296–297.
7. Adson, M.A. & Farnell, M.B. (1981) Hepatobiliary cancer—surgical considerations. *Mayo Clinic Proceedings*, **56**, 686–699.
8. Bismuth, H., Houssin, D. & Michel, F. (1983) Le risque operatoire des hépatectomies. *Experiences sur 154 hépatectomies. Chirurgie*, **109**, 342–348.
9. Ekberg, H., Tranberg, K.G., Andersson, R., Lundstedt, C., Hägerstrand, I., Ranstam, J. & Bengmark, S. (1986) Determinants of survival in liver resection for colorectal secondaries. *British Journal of Surgery*, **73**, 727–731.
10. Fortner, J.G., Kim, D.K., Maclean, B.J., Barrett, M.K., Iwatsuki, S., Turnbull, A.D., Howland, W.S. & Beattie, E.J. (1978) Major hepatic resection for neoplasia: personal experience in 108 patients. *Annals of Surgery*, **188**, 363–371.
11. Fortner, J.G., Maclean, B.J., Kim, D.K., Howland, W.S., Turnbull, A.D., Goldiner, P., Carlon, G. & Beattie, E.J. (1981) The seventies evolution in liver surgery for cancer. *Cancer*, **47**, 2162–2166.
12. Gunvén, P., Makuuchi, M., Takayasu, K., Moriyama, N., Yamazaki, S. & Hasegawa, H. (1985) Preoperative imaging of liver metastases. Comparison of angiography, CT scan, and ultrasonography. *Annals of Surgery*, **202**, 573–579.
13. Shimamura, Y., Gunvén, P., Takenaka, Y., Shimizu, H., Shima, Y., Akimoto, H., Arima, K., Takahashi, A., Kitaya, T., Matsuyama, T. & Hasegawa, H. (1988) Combined peripheral and central chemoembolization of liver tumors. Experience with Lipiodol—doxorubicin and gelatin sponge (L-TAE). *Cancer*, **61**, 238–242.
14. Stone, H.H. (1988) Trauma to the liver vasculature. Aneurysm and arteriovenous fistula. In: Blumgart, L.H., ed. Surgery of the liver and biliary tract, vol. **2**. Edinburgh, London, Melbourne, New York: Churchill Livingstone, pp 1068–1069.
15. Blumgart, L.H. (1988) Liver resection—liver and biliary tumours. In: Blumgart, L.H., ed. Surgery of the liver and biliary tract, vol. **2**. Edinburgh, London, Melbourne, New York: Churchill Livingstone, pp 1272–1273.

*(Accepted by S. Bengmark 13 March 1989)*

